# LAight® Therapy Is an Effective Treatment Option to Maintain Long-Term Remission of Hurley I and II Hidradenitis Suppurativa: Results from Period B of RELIEVE, a Multicenter Randomized, Controlled Trial

**DOI:** 10.1159/000524739

**Published:** 2022-06-09

**Authors:** Michael Schultheis, Petra Staubach, Stephan Grabbe, Christian Ruckes, Esther von Stebut, Uwe Kirschner, Łukasz Matusiak, Jacek C. Szepietowski, Georgios Nikolakis

**Affiliations:** ^a^Department of Dermatology, University Medical Center, Johannes Gutenberg University, Mainz, Germany; ^b^Interdisciplinary Centre for Clinical Studies, University Medical Center, Johannes Gutenberg University, Mainz, Germany; ^c^Department of Dermatology, Faculty of Medicine, University of Cologne, Cologne, Germany; ^d^Dermatology Outpatient Office Dr. Uwe Kirschner, Mainz, Germany; ^e^Department of Dermatology, Venereology and Allergology, Wroclaw Medical University, Wroclaw, Poland; ^f^Departments of Dermatology, Venereology, Allergology and Immunology, Dessau Medical Center, Brandenburg Medical School Theodor Fontane and Faculty of Health Sciences Brandenburg, Dessau, Germany

**Keywords:** Hidradenitis suppurativa, LAight®, Topical treatment, Noninvasive device

## Abstract

**Background:**

Hidradenitis suppurativa is a chronic, inflammatory, burdensome skin disease where current first-line treatments are limited to topical and/or systemic antibiotics which cannot be applied for long-term disease management. Period B of the RELIEVE study analyzes whether LAight® therapy can sustain or even increase remission after a first topical antibiotic treatment cycle.

**Methods:**

The RELIEVE study was performed as a two-period multicenter randomized controlled trial with blinded assessment. For period A from week 0 to week 16, the 88 participating Hurley I and II patients were randomized to either a group receiving topical clindamycin 1% solution combined with 8 additional bi-weekly treatments with LAight® therapy (group TC + L) or a group which was treated with topical clindamycin 1% solution only (group TC). After 16 weeks, patients entered open-label period B and both groups were treated exclusively with LAight® therapy for an additional 16 weeks (8 sessions, group TC + L/L and group TC/L).

**Results:**

In total, 88 patients were enrolled in RELIEVE. Seventy-eight patients entered period B; 39 belonged to group TC + L/L and 39 to group TC/L. The IHS4-response at the start of period B was 62% (group TC + L/L) and 33% (group TC + L). During the 16 weeks of additional monotherapy with LAight, in both groups >90% of patients who responded to therapy in period A maintained their IHS4-response at week 32. IHS4 response rates continued to rise up to 79% of the TC + L/L group and up to 71% of the TC/L group during period B at week 32. Achievement of HiSCR and certain patient reported outcomes confirmed primary endpoint results.

**Conclusion:**

LAight® therapy is an effective approved therapy option for Hurley I and II HS that can be used continuously to maintain treatment success. During 16 weeks of follow-up in period B, over 90% of patients with response after period A maintained their treatment outcome, while more than 60% of prior nonresponders gained response. The fact that LAight® therapy can be applied continuously, is very effective and is well tolerated makes it a valuable treatment tool in the design of HS long-term treatment modalities.

## Introduction

Hidradenitis suppurativa (HS), also known as acne inversa, is a debilitating, chronic skin condition characterized by recurrent episodes of inflammation associated with the formation of abscesses, inflammatory nodules, pain, and drainage ultimately culminating in the formation of scarring in moderate to severe disease. The axillae, breasts, groin, buttocks, and lower abdomen are common intertriginous regions affected by HS.

According to the European guidelines, the medical repertoire of first-line treatments includes topical and oral antibiotics [1, 2]. Those therapies are mostly off-label and cannot be used as long-term treatment. So, even though they can successfully control symptoms, discontinuation is often associated with relapses [3, 4, 5]. The fact that HS is a chronic disease imposes challenges on establishing long-term treatment plans since it is known that repeated treatment cycles with antibiotics may induce antibiotic resistance in patients [6].

For moderate to severe disease, continuous treatment with adalimumab as biologic therapy is applicable after failed systemic antibiotic treatment. Despite the overall good safety profile of this biologic, significant side effects have been described [7, 8]. When applied in daily practice, besides the associated contraindications for treatment [9], the formation of antibodies [10] and the significant costs of the biologic treatment might also put a strain on its long-term application. Therefore, there is a medical need for cost efficient and well-tolerated treatment options with little contraindications which can be applied long-term to all disease severities and may prevent progression or relapses of the disease.

The LAight® therapy (LENICURA, Germany) is performed by using a noninvasive device which is CE-approved for all degrees of severity of HS. It utilizes a combination of radiofrequency (RF) and intense pulsed light (IPL). The results of the primary endpoint analysis of period A of the RELIEVE study showed that a combined therapy with LAight® and topical clindamycin 1% solution reduced disease severity and improved quality of life significantly more effectively than clindamycin alone [11].

In the following, the results of period B of the RELIEVE study are presented to assess whether LAight® therapy can sustain remission in prior treatment responders. Additionally, it will be evaluated whether LAight® therapy can lead to further IHS4 reduction beyond treatment week 16, after initially failed therapy, i.e., nonresponders, with topical clindamycin 1% solution only (TC/L) or combined LAight® therapy and clindamycin therapy (TC + L/L).

## Materials and Methods

### Patients

**Fig. 1:**
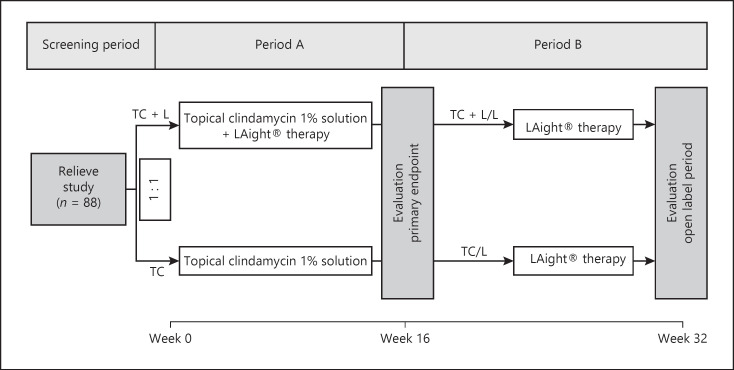
RELIEVE study design. The work at hand does report the results of period B, while period A outcomes were already reported in a preceding paper.

Patients were recruited from June 26, 2019, until June 26, 2020, in the four participating centers (Department of Dermatology, University Medical Center, Johannes Gutenberg University, Mainz, Germany; Departments of Dermatology, Venereology, Allergology and Immunology, Dessau Medical Center, Brandenburg Medical School Theodor Fontane, Dessau, Germany; Dermatology Outpatient Office Dr. Uwe Kirschner, Mainz, Germany; Department of Dermatology, Venereology and Allergology, Wroclaw Medical University, Wroclaw, Poland). Recruiting was completed as soon as 88 patients had been enrolled. The study design and details are shown in Figure 1.

Patients were eligible for study participation if they were diagnosed with HS, were of legal age, suffered from Hurley stage I or II disease, and presented with at least one affected area typical for HS (axillary, inguinal, gluteal, mammary) at baseline. Moreover, at time of inclusion patients must have had at least 3 inflammatory nodules or abscesses as well as the mental ability to understand the patient information and follow the study procedure.

Patients were excluded if they suffered from Hurley stage III HS, showed contraindications toward topical clindamycin 1% solution or LAight® therapy, had other diseases that can lead to systemic inflammation, were treated with oral antibiotics or retinoids within the last 2 weeks, received a biologic treatment during the prior 6 months, or already had a LAight® therapy session in the past. All patients provided written consent before randomization.

### Study Design

The RELIEVE study was performed as a two-phase multicenter randomized controlled trial with blinded assessment. It was conducted in accordance with the WHO Declaration of Helsinki and approved by the local Ethics Committees. The trial was registered with the German Register for Clinical Trials (DRKS, # DRKS00017543).

For period A from week 0 to week 16, participating subjects were randomized to either a group receiving topical clindamycin 1% solution combined with 8 additional bi-weekly treatments with LAight® therapy (group TC + L) or a group which was treated with topical clindamycin 1% solution only (group TC). After 16 weeks, patients entered open-label period B and both groups were treated exclusively with LAight® therapy for an additional 16 weeks (8 sessions, group TC + L/L and group TC/L).

The full LAight® therapy includes three treatment passes combining IPL and bipolar nonfractional RF differing with respect to applied cut-off values for the IPL pulses; details are presented in Table 1.

Throughout period B, the following termination criteria were applied: surgical interventions beyond a single incision and development of contraindications to LAight® therapy (e.g., pregnancy), as well as an interval between individual sessions of <10 days or >18 days. The contraindications of the LAight® therapy, adverse events and/or additional therapeutic interventions, were checked before each session, along with specific possible adverse events of the therapy.

### Assessments

Assessments took place at week 0, 8, 16, 24, and 32. The medical assessments of disease severity were carried out by an unblinded investigator and were additionally repeated by a dermatologist blinded with respect to study allocation. The number of active inflammatory HS lesions (inflammatory nodules, abscesses, and draining fistulas), serving as basis for the clinical scores, was counted considering both visible and palpable lesions. In addition, photographic documentation was performed, and questionnaires were handed out to patients on-site. Moreover, patients were asked about their treatment experience after every LAight® therapy, where the scale was defined as follows: no pain = 0, slight pain = 1, moderate pain = 2, severe pain = 3, very severe pain = 4, and strongest imaginable pain = 5.

### Follow-Up Analysis

**Fig. 2:**
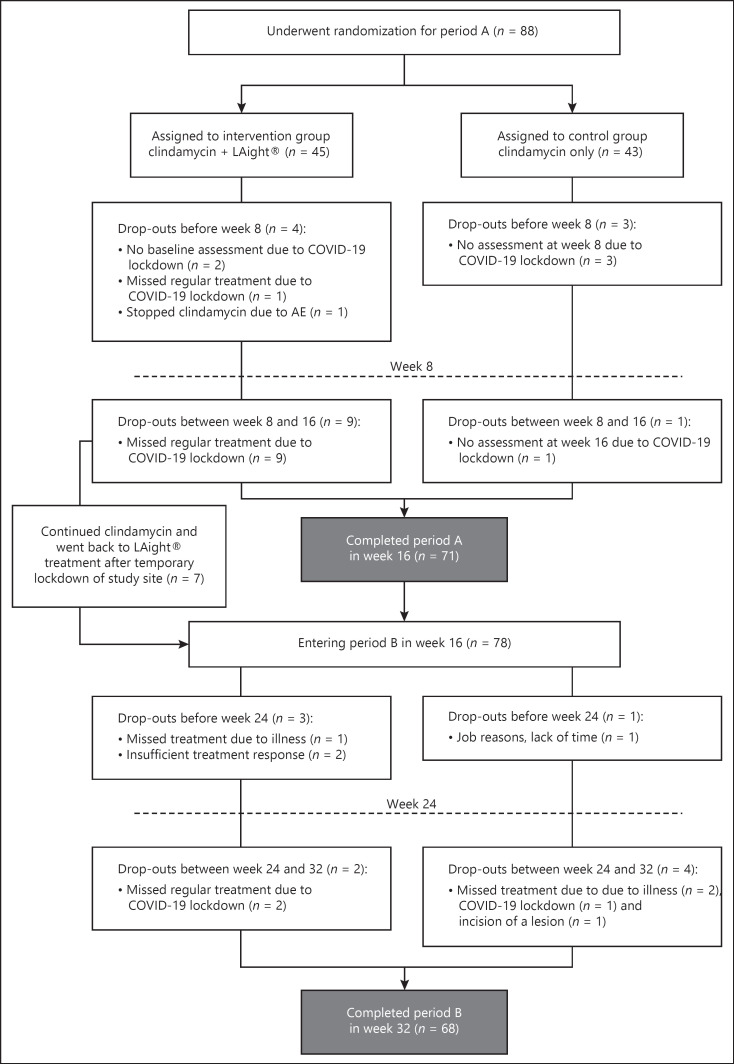
CONSORT flow diagram.

The data evaluation was carried out by the Interdisciplinary Centre for Clinical Studies Mainz as an independent institution. All 78 patients entering period B were included in the follow-up analysis (see Fig. 2). The reported values are based on the assessment of the blinded evaluator.

To analyze whether LAight® therapy can stabilize treatment effects, response rates on the scores applied in period A were used. Those included the clinical International Hidradenitis Suppurativa Score System (IHS4) [12], the Hidradenitis Suppurativa Clinical Response (HiSCR) [13], and the DLQI [14] as well as the assessment of pain during the last 24 h on the numeric rating scale (NRS) [15] as patient reported outcomes.

Responder values were calculated in the per-protocol analysis with respect to baseline values by utilizing the software SAS (SAS, Cary, NC, USA) and the following definitions of response with respect to scores named above: response in IHS4 is achieved if the decrease in the score is ≥55% [16]. HiSCR is defined as a ≥50% reduction in inflammatory lesion count (sum of abscesses and inflammatory nodules) and no increase in abscesses or draining fistulas [13]. For the DLQI, Basra et al. [17] established a minimal clinically important difference for inflammatory skin diseases. Thus, patients with a DLQI reduction of ≥4 points are considered to be responders (including only patients with baseline values of ≥4 points). Patients with pain score of ≥3 at baseline and a reduction of ≥30% and ≥1 unit in pain score are defined as responders [18]. If the data were normally distributed, the two-sided paired *t* test was used; otherwise, the Wilcoxon signed-rank test was applied. Confidence level was set to 5%.

This paper describes results of period B of the RELIEVE study. Findings of period A investigating the efficacy of LAight® treatment combined with first-line medical therapies in Hurley I and II are presented in a precedent paper [11].

## Results

### Study Participants

In total, 88 patients were enrolled in RELIEVE (Fig. 2). Baseline characteristics, including the risk factors smoking and obesity, were similar between the two groups, although patients in the TC + L group were slightly more heavily affected than those in the TC group (Table 2). One study site had to temporarily close due to COVID-19 during period A. Therefore, 7 patients from the TC + L group did not receive all 8 treatments during period A (3 treatments missed) and were excluded for evaluation in the primary endpoint analysis of period A. However, due to ethical consideration the site continued assessments and treatment of those patients and all 7 patients received the full 8 treatments in period B. The authors decided to include those patients as members of the TC + L/L in period B (Fig. 2) since all assessments were available, patients continued clindamycin during period A, had 5 treatments with LAight® during the first 16 weeks, and received all treatments after cross over to period B. Therefore, for period B, 78 patients are evaluated of which 39 were previously treated in the TC + L group and 39 in the TC group.

### Outcome Analysis

**Fig. 3:**
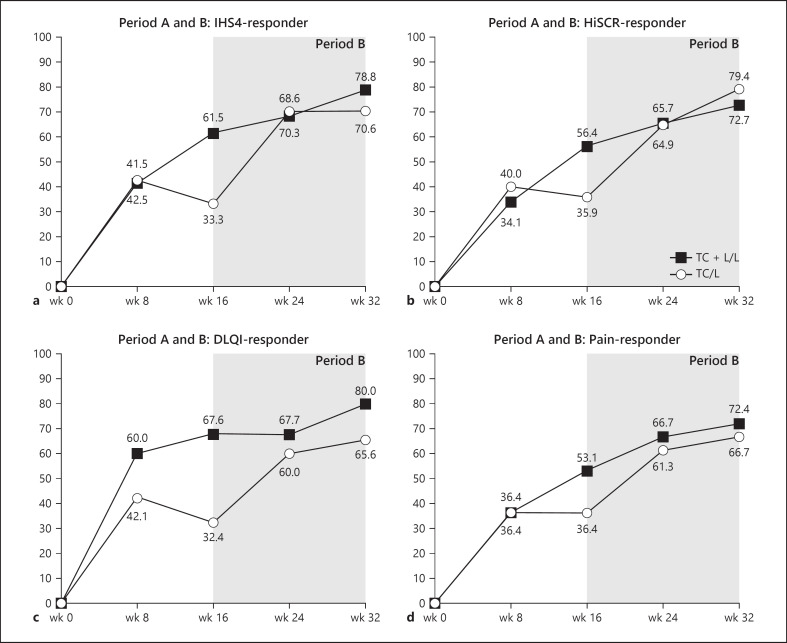
**a–d** Development of response rates in IHS4, HiSCR, DLQI, and pain-NRS from baseline to week 32 (due to the inclusion of the additional seven period A drop-outs in the period B analysis, values in week 8 and 16 of the TC + L/L slightly differ from the reported endpoint analysis in the period A publication [22]).

Concerning clinical scores, at the start of open-label period B, the TC + L group showed a 61.5% response rate in IHS4 and a 56.4% response rate in HiSCR, while the values for the TC group were 33.3% and 35.9%, respectively. After 8 more treatments with LAight® therapy, those values increased for the TC + L/L group to a 78.8% response rate in IHS4 and a 72.7% response rate in HiSCR, while for the TC/L group response rates more than doubled (70.6% in IHS4 and 79.4% in HiSCR) (Fig. 3a, b).

**Fig. 4:**
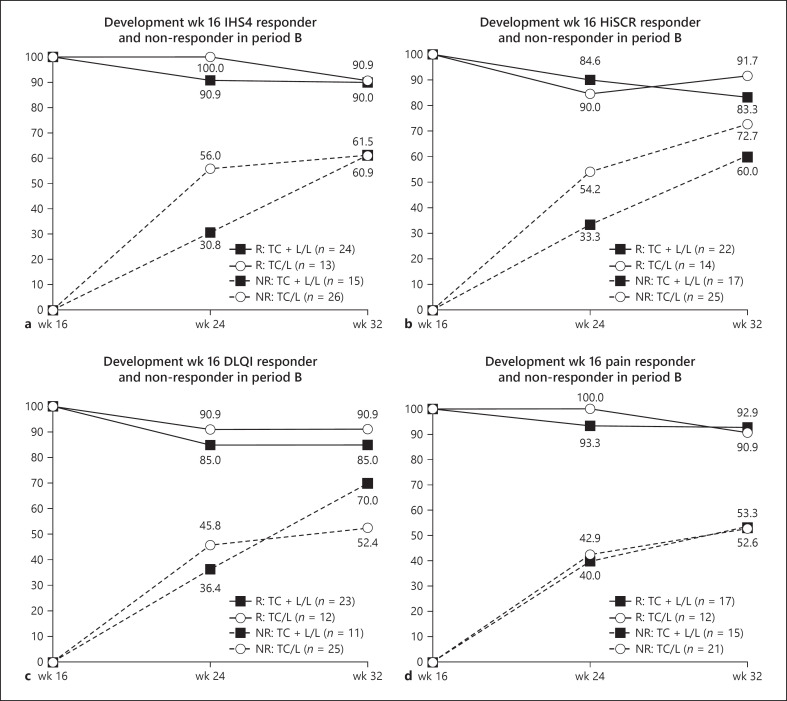
**a–d** Development of response rates in IHS4, HiSCR, DLQI, and pain-NRS from week 16 to week 32; for week-16 responders (R) starting at 100% and week-16 nonresponders (NR) starting at 0%.

After 16 weeks of additional monotherapy with LAight®, period A IHS4-response could be maintained for 90.9% of patients previously treated with the combination of LAight® therapy and topical clindamycin 1% solution but also for 90% of the patients achieving response with the topical antibiotic only (83.3% and 91.7% for HiSCR). Of the IHS4 nonresponders at end of period A, 61.5% of the TC + L/L group and 60.9% of the TC/L group gained response until week 32. For the HiSCR, the corresponding values were 60% and 72.7%, respectively (Fig. 4a, b). The development of the patient reported outcomes, DLQI and pain-NRS, showed a similar picture with high maintenance of week-16 response and an increase in overall response rates until week 32 (Fig. 3c, d, Fig. 4c, d).

**Fig. 5:**
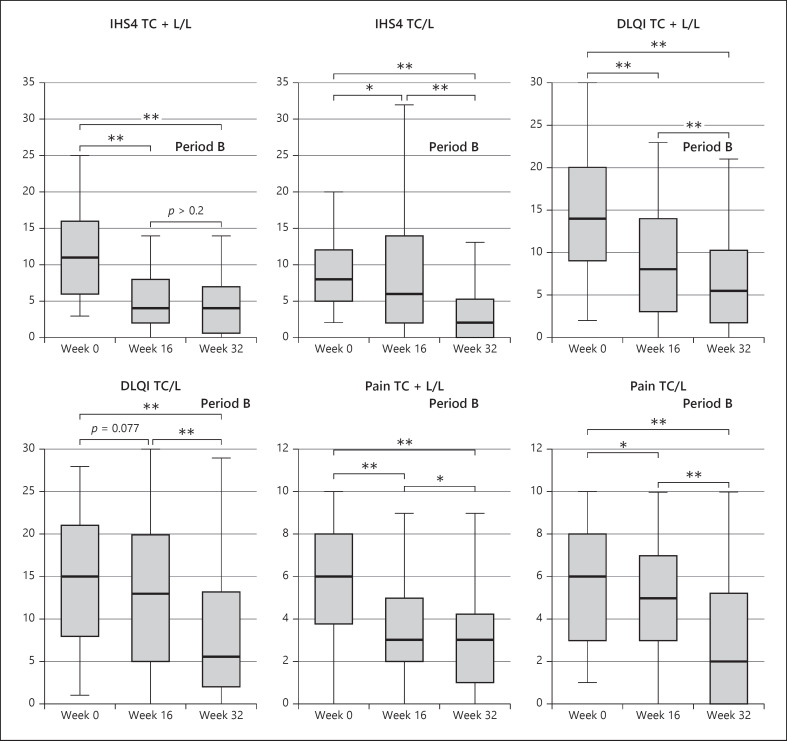
Development of median in IHS4, DLQI, and pain-NRS from baseline to week 32; where * stands for a *p* value ≤0.05 and ** stands for a *p* value ≤0.01 when applying the Wilcoxon signed-rank test (due to the inclusion of the additional seven period A drop-outs in the period B analysis, values in week 8 and 16 of the TC + L/L slightly differ from the reported endpoint analysis in the period A publication [22]).

**Fig. 6:**
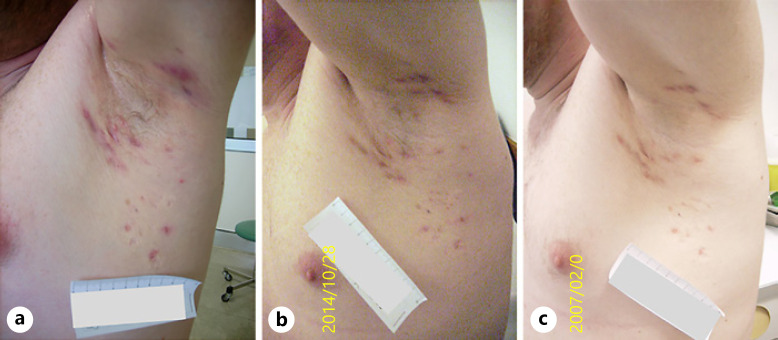
Manifestation of HS lesions in the right axilla of a participant of the TC + L/L at baseline (**a**), at time of primary endpoint evaluation at week 16 (**b**), and after open-label period B at week 32 (**c**).

A median patient entering the treatment algorithm of the TC + L/L group experiences a decrease in IHS4 of 63.6% (11 points to 4 points, *p* < 0.001) during the first 16 weeks of combined treatment which can be maintained for additional 16 weeks while quality of life also significantly improves from 14 points at baseline to 8 points at week 16 (*p* < 0.001) to finally 5.5 points in week 32 (a total change of −60.7%, *p* < 0.001), while pain decreases from 6 points to 3 points (−50%, *p* < 0.001) (Fig. 5, 6). Put into perspective, this implies that the TC + L/L group protocol transforms severe disease into almost mild disease and changes the very large effect on patient's life into an only small effect on patient's life.

**Fig. 7:**
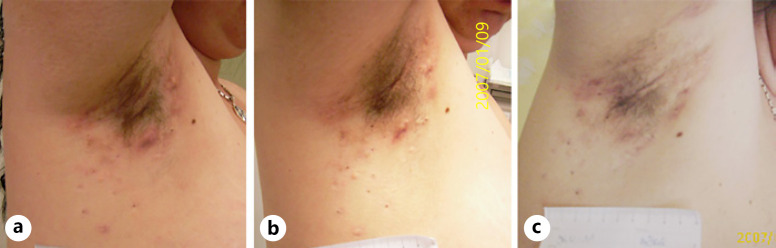
Manifestation of HS lesions in the right axilla of a participant of the TC/L at baseline (**a**), at time of primary endpoint evaluation at week 16 (**b**), and after open-label period B at week 32 (**c**).

The IHS4 of a median patient entering the treatment algorithm of the TC/L group decreases by 25% (8 points to 6 points, *p* = 0.013) during the first 16 weeks and is again markedly reduced when treated for additional 16 weeks with LAight® therapy (6 points to 2 points; *p* < 0.001). The analysis of the development of quality of life supports the impression of the clinical endpoint since DLQI improved by 13.3% (15 points to 13 points, *p* = 0.077) in period A and again by 7.5 points until week 32 to a final level of 5.5 points (*p* < 0.001) (Fig. 5, 7).

### Safety and Tolerance

During period B of the RELIEVE study, a total of 530 sessions with LAight® therapy were performed during which 13 out of 78 patients (16.7%) noted 47 adverse events (Table 3). As in period A, erythema was the most common side effect and all reported side effects were of temporary nature. During period A, 27.9% of patients had reported side effects which is more than during period B. This is in line with the average level of pain of 1.0 ± 1.00 noted during therapy in period B, which was significantly lower than the reported 1.4 ± 1.15 in period A (*p* < 0.001, *t* test).

There were 2 adverse events recorded by the responsible dermatologist during assessment at week 24, both strong itching (*n* = 1, TC + L/L group; *n* = 1, TC/L group). There was no adverse event documented during the final assessment in week 32.

## Discussion

HS is a chronic inflammatory disease with a high burden. Therapeutic options exist − however, studies show that those do not always fit patient needs, mainly due to poor efficacy, relapses, and undesirable side effects [19]. The results of period A of the RELIEVE study show that the combination of LAight® therapy with topical clindamycin 1% solution for 16 weeks is an effective and safe approach to treat Hurley stage I and II HS. The combination was clearly dominant over monotherapy with topical clindamycin 1% solution alone [11]. Period B of the presented RELIEVE study evaluated whether LAight® treatment as monotherapy has the potential to maintain, or even improve, therapeutic effects beyond treatment week 16 after initially failed therapy, i.e., nonresponders, with topical clindamycin 1% solution only (TC/L) or combined LAight® therapy and clindamycin therapy TC + L/L.

In current guidelines, monotherapy with topical clindamycin 1% solution is suggested for mild, localized disease while systemic antibiotic therapy (tetracyclines, clindamycin, and rifampicin) is currently first-line therapy for moderate widespread HS with rather subcutaneous lesions [20]. An RCT compared clindamycin 1% solution against tetracycline 500 mg topical in 46 patients with Hurley stage I and II HS and found no significant differences between groups [5]. In patients with moderate-to-severe HS, guidelines recommend systemic clindamycin plus rifampicin as the first-line treatment option. Interestingly, a recent study by van Straalen et al. [21] also showed no significant differences between clindamycin plus rifampicin and tetracycline for the validated outcomes HiSCR, pain, or DLQI, for all severities of HS. Hence, for 12 weeks of treatment of Hurley I and II, there does not seem to be much of a difference between the suggested first-line antibiotic options. Moreover, the long-term combination of clindamycin with rifampicin did not provide an additional benefit in terms of IHS4 and DLQI reduction after a 5-day iv clindamycin treatment in a recent study [22]. Van Straalen et al. [21] reported the response rates on outcomes for all Hurley stages separately and it can be noted that the combination of LAight® therapy with topical clindamycin 1% solution for 16 weeks achieves higher response rates in HiSCR and DLQI and a slightly lower response rate in pain than both antibiotic treatment schemes with respect to the comparable patient groups Hurley I and II.

Guidelines recommend stopping treatment with antibiotics after latest 16 weeks and to restart therapy when necessary [12]. Even if there are publications on safety of prolonged antibiotic therapy in HS [23], the public health importance needs to be considered. The World Health Organization describes antimicrobial resistance as one of the biggest threats to global health today and recommends that antibiotics are prescribed only when needed. Due to the long-term administration of antibiotics, already up to 65% of bacterial cultures in patients with HS are resistant to clindamycin [24]. Long-term administration of rifampicin and its reduced level in the blood after combination with clindamycin were also criticized [23, 25, 26].

With respect to relapses, there are not much data available on antibiotic therapies in HS. Among 6 case series on clindamycin plus rifampicin including in total 178 patients, one series reported a 0% recurrence rate, and two studies showed a 59–61.5% relapse 4–5 months after stopping treatment [20]. The results of period B of RELIEVE presented above showed that during the 16 weeks of additional monotherapy with LAight, in both groups >90% of patients who responded to therapy in period A maintained their IHS4-response at week 32. Of the IHS4 nonresponders at end of period A, 61.5% of the TC + L/L group and 60.9% of the TC/L group gained response until week 32. So, overall IHS4 response rates continued to rise to 79% during period B in patients of the TC + L/L group while patients who only received topical clindamycin beforehand (group TC/L) rapidly responded to LAight® therapy during period B, reaching almost comparable response rates of 71% at week 32.

While there is no promising medical option for long-term therapy for mild disease, adalimumab can be administered for moderate to severe disease after inefficient antibiotic treatment. In both PIONEER trials, response rates for week 12 responders could be maintained for approximately 50% until week 32 while around 40% of nonresponders gained response with weekly injections [18]. Those rates are evidently lower than those under monotherapy with LAight® until week 32, which speaks for the application of LAight® therapy as continuous therapy for Hurley I and II patients. Moreover, a recent case study showed that LAight® therapy can also prevent relapses after termination of adalimumab in Hurley stage III disease [27].

The high effectivity in prevention of flares might stem from the physical properties of the LAight® therapy. It is known that RF induces collagen production, increases blood flow in the treated area, and mediates liquification of enclosed lipids, releasing hair follicle blockage [28]. The decrease of follicular inclusion, which is still considered the primary event in the formation of HS lesions, might already stop the process prior to secondary infection and the release of pro-inflammatory cytokines.

All side effects associated with LAight® treatment in both periods of the RELIEVE study were mild and temporary. However, during period A, more patients (27.9%) reported those effects than during period B (16.7%). This is in line with the average level of pain of 1.0 ± 1.00 noted during therapy, which was significantly lower during period B than the reported level of 1.4 ± 1.15 in period A (*p* < 0.001, *t* test). Therefore, monotherapy with LAight® is tolerated even better than when combined with topical clindamycin 1% solution. However, this might be a result of the overall lower level of inflammation at start of period B in comparison to baseline. Especially when compared with all other current treatment options, LAight® possesses a significantly better overall side effect profile, as gastrointestinal, severe neurological, or rheumatic processes cannot be induced.

To conclude, LAight® treatment in combination with topical clindamycin 1% solution was proven to be an effective and safe treatment option for Hurley I and II patients. Moreover, LAight® therapy as monotherapy was able to maintain high response rates after termination of the antibiotic and led to further IHS4 reduction beyond treatment week 16 for patients with initially failed therapy, i.e., nonresponders, with topical clindamycin 1% solution only or combined LAight® therapy and clindamycin therapy. The treatment can be performed in an outpatient setting at a doctor's office by the physician or trained nurse. The fact that LAight® therapy can be applied to all disease severities has only mild and transitory side effects and is very cost effective that makes it a valuable component in the design of HS-long-term treatment plans.

## Key Message

LAight® can successfully retain remission in Hurley stage I and II HS.

## Statement of Ethics

The research complied with the guidelines for human studies and was conducted ethically in accordance with the World Medical Association Declaration of Helsinki. The study protocol was approved by the institute's committee on human research and was registered prior to recruitment in a viable clinical trial register (German Register for Clinical Trials; # DRKS00017543).

## Conflict of Interest Statement

Michael Schultheis: speaker for AbbVie. Petra Staubach: advisor/consultant: AbbVie, Allergika, Almirall Hermal, Amgen, Beiersdorf, Biocryst, Biogen Idec, BMS, Boehringer Ingelheim, Celgene, CSL Behring, Eli Lilly, Galderma, Hexal, Janssen, Klinge, Klosterfrau, LEO Pharma, LETI Pharma, L'Oreal, Novartis, Octapharma, Pfizer, Pflüger, Pharming, Regeneron, Shire, Takeda, Regeneron, Sanofi-Genzyme, und UCB Pharma.

Stephan Grabbe: advisor/consultant: AbbVie (also travel funding), BMS (also travel funding), MSD (also travel funding), SUN Pharma, Roche (also travel funding), Pfizer, Sanofi Pasteur MSD, Takeda, Novartis, Merck, Guidepoint Global, and Merck Serono and research support: Novartis and Pierre Fabre. Georgios Nikolakis and Christian Ruckes: nothing to declare.

Esther von Stebut: speaker for Novartis, Janssen, and LEO Pharma. Uwe Kirschner: speaker for/consultant to Mylan, Germany, and travel funding: LENICURA.

Jacek C. Szepietowski: advisor/consultant for AbbVie, Clexio Biosciences, LEO Pharma, Menlo Therapeutics, Novartis, Pierre Fabre, Sanofi-Genzyme, Trevi, and Viofor and speaker for AbbVie, Eli Lilly, Janssen-Cilag, LEO Pharma, Novartis, Sanofi-Genzyme, and SunFarm. Lukasz Matusiak: advisor/consultant for AbbVie, LEO Pharma, and Pierre Fabre and speaker for AbbVie, Pierre Fabre, Valeant, and SunFarm.

## Funding Sources

The LENICURA GmbH provided the treatment device to two centers (Wroclaw and Dessau), provided support, training, and infrastructure free of charge, and contributed the contact gel for treatment. This work was supported in part by Biomolecular Analyses for Tailored Medicine in AcneiNversa (BATMAN) project, funded by ERA PerMed (to E.v.S.).

## Author Contributions

Michael Schultheis, Petra Staubach, Stephan Grabbe, Christian Ruckes, Esther von Stebut, Uwe Kirschner, Łukasz Matusiak, Jacek C. Szepietowski, and Georgios Nikolakis:

1. Substantial contributions to the conception or design of the work, or the acquisition, analysis, or interpretation of data for the work.
2. Drafting the work or revising it critically for important intellectual content.
3. Final approval of the version to be published.
4. Agreement to be accountable for all aspects of the work in ensuring that questions related to the accuracy or integrity of any part of the work are appropriately investigated and resolved.

## Data Availability Statement

All data generated or analyzed during this study are included in this article. Further inquiries can be directed to the corresponding author.

## Figures and Tables

**Table 1:** Technical characteristics of LAight^®^ therapy

Treatment passes	IPL wavelength interval in nm	IPL intensities in J/cm^2^	Impulse characteristics IPL	RF intensities in J/cm^2^	Impulse characteristics RF
First treatment pass	420–1,200	6.0	4 sub-impulses with 8-ms duration and 8-ms pause		1 impulse with 1-s duration and frequency of 1 MHz

Second treatment pass	510–1,200	5.6		12.2	

Third treatment pass	690–1,200	4.4			

**Table 2:** Patient characteristics

Characteristics	LAight and clindamycin	Clindamycin only
Patients, *n*	43	43
Female sex, *n* (%)	23 (53.5)	24 (55.8)
Age in years − mean ± SD	37.9±11.36	37.5±12.86
Disease duration in years − mean ± SD	14.2±10.59	13.1±7.47
Time to diagnosis in years − mean ± SD	10.1±11.00	8.0±7.84
Disease severity		
Hurley I, *n* (%)	13 (30.2)	17 (39.5)
Hurley II, *n* (%)	30 (69.8)	26 (60.5)
Areas affected − mean ± SD	4.6±3.06	3.7±2.48
Inflammatory nodules − mean ± SD	7.0±6.41	5.6±5.06
Abscesses − mean ± SD	0.9±1.60	0.9±1.74
Draining fistulas − mean ± SD	0.7±1.09	0.7±1.44
IHS4 − mean ± SD	11.7±7.10	10.2±8.72
PROs		
DLQI − mean ± SD	14.1±7.13	14.6±7.34
Pain NRS − mean ± SD	5.8±2.83	5.8±2.77
HADS − mean ± SD	15.5±7.99	15.1±8.85
Risk factors and skin type		
Smoker, *n* (%)	24 (55.8)	26 (60.5)
Cigs. per day − mean ± SD	7.0±7.53	9.7±8.98
BMI − mean ± SD	30.3±6.42	32.0±8.21
Skin type according to F.P., *n* (%)		
Skin type 1	1 (2.3)	5 (11.6)
Skin type 2	32 (74.4)	26 (60.5)
Skin type 3	7 (16.3)	9 (20.9)
Skin type 4	1 (2.3)	3 (7.0)
Skin type 5	0 (0.0)	0 (0.0)
Previous treatments		
Clindamycin (topical), *n* (%)	12 (27.9)	8 (18.6)
Duration in weeks − mean ± SD	6.5±5.14	7.5±5.13
Clindamycin + rifampicin (oral), *n* (%)	17 (39.5)	13 (30.2)
Duration in weeks − mean ± SD	8.3±6.16	7.2±6.73
Retinoids, *n* (%)	10 (23.3)	6 (14.0)
Duration in weeks − mean ± SD	14.4±13.79	14.3±17.08
Other, *n* (%)	13 (30.2)	12 (27.9)
Duration in weeks − mean ± SD	7.3±6.73	9.8±6.53
Prior surgery for HS		
Patients with incision, *n* (%)	25 (58.1)	19 (44.2)
Incisions, *n* − mean ± SD	3.4±4.79	2.8±2.57
Patients with deroofing, *n* (%)	2 (4.7)	0 (0.0)
Deroofing, *n* − mean ± SD	1.5±0.71	0±0.00
Patient with excisions, *n* (%)	13 (30.2)	22 (51.2)
Excisions, *n* − mean ± SD	2.5±1.81	3.3±5.43

**Table 3:** Adverse events during period B

Adverse event	Erythema	Edema/swelling	Blisters/crustification	Pigment change	Wound infection	Other AE during therapy
Events, *n*	26	18	1	0	0	2*
% of 530 sessions total number of events	4.9	3.4	0.2	0.0	0.0	0.6

* Itching (*n* = 2).
